# Disturbed macro-connectivity in schizophrenia linked to oligodendrocyte dysfunction: from structural findings to molecules

**DOI:** 10.1038/npjschz.2015.34

**Published:** 2015-09-23

**Authors:** Juliana Silva Cassoli, Paul C Guest, Berend Malchow, Andrea Schmitt, Peter Falkai, Daniel Martins-de-Souza

**Affiliations:** 1 Laboratory of Neuroproteomics, Department of Biochemistry and Tissue Biology, Institute of Biology, University of Campinas (UNICAMP), Campinas, Brazil; 2 Department of Psychiatry and Psychotherapy, Ludwig-Maximilians-University (LMU), Munich, Germany; 3 Laboratory of Neurosciences (LIM-27), Institute of Psychiatry, University of São Paulo (USP), São Paulo, Brazil; 4 UNICAMP’s Neurobiology Center, Campinas, Brazil

## Abstract

Schizophrenia is a severe psychiatric disorder with multi-factorial characteristics. A number of findings have shown disrupted synaptic connectivity in schizophrenia patients and emerging evidence suggests that this results from dysfunctional oligodendrocytes, the cells responsible for myelinating axons in white matter to promote neuronal conduction. The exact cause of this is not known, although recent imaging and molecular profiling studies of schizophrenia patients have identified changes in white matter tracts connecting multiple brain regions with effects on protein signaling networks involved in the myelination process. Further understanding of oligodendrocyte dysfunction in schizophrenia could lead to identification of novel drug targets for this devastating disease.

## Introduction

Schizophrenia (SCZ) is a group of severe psychiatric disorders with lifelong disability occurring in >50% of the sufferers, making it one of the 10 most costly illnesses worldwide.^1^ The course of the disease is heterogeneous and characterized variously by the well-known positive symptoms such as psychosis, hallucinations, and delusions, as well as negative symptoms and cognitive deficits.^2^ Despite recent advances leading to new scientific insights into this disorder, consistent neurobiological markers for SCZ are lacking and diagnosis still relies on subjective assessment of a cluster of signs and symptoms, based on psychiatric rating systems such as the International Statistical Classification of Diseases and Related Health Problems 10th Revision and the Diagnostic and Statistical Manual of Mental Disorders, Fifth Edition.^3^ Treatment with antipsychotics helps to relieve some of the positive symptoms, although this has little or no effect on the negative symptoms or cognitive deficits, and most patients continue to suffer from these throughout their lifetimes.^4,5^

Considerable efforts are now underway using imaging and biomarker studies, which have marginally increased our understanding of the neurobiological basis of the disease. It is anticipated that further efforts in this area will lead to improved diagnosis or evaluation of the course of the disease and may also lay the groundwork for the development of new innovative treatment strategies. The main findings of these studies have led to the concept that the neurological deficits arise from an interaction between genetic^6^ and environmental factors.^7^ This result in SCZ symptoms that emerge during early adulthood and associated structural alterations in specific brain regions, leading to dysfunctional neuronal circuits and impaired connectivity through effects on white matter in complex brain networks.^8–10^

This review details the latest findings concerning the role of oligodendrocytes in the neuronal disconnectivity in SCZ from studies that have used imaging and biomarker profiling approaches. Most importantly, it will highlight how further studies along these avenues will result in increased understanding of the pathways affected in this devastating disease as well as the identification of much-needed novel drug targets for improved patient outcomes.

## Schizophrenia—a result of brain disconnectivity?

One of the most recurrent findings has implicated disrupted intra- and inter-region connectivity as being the cause of many hallmark symptoms of SCZ. This is because normal brain function requires coordinated function of multiple brain regions in tasks such as perception and cognition, as well as for emotions and mood responses. Disconnectivity has been demonstrated in fronto-temporal regions,^11^ cortico-thalamo-cerebellar loops,^12^ and inter-hemispheric fibers crossing in the corpus callosum.^13^ A meta-analysis of 15 voxel-based diffusion tensor imaging studies revealed reduced fractional anisotropy as a measure of fiber density, myelination, and intra-tract coherence in left frontal and temporal lobe white matter in SCZ patients. These findings point towards disconnectivity in two distinct white matter tracts, one linking the frontal cortex, thalamus, and cingulate gyrus and the other forming a connection between the frontal cortex, insula, hippocampus, and temporal cortex.^14^ However, as chronic patients were used in these studies, it is possible that antipsychotic treatment was a confounding factor. Nevertheless, a recent meta-analysis of first episode patients with only marginal treatment also showed a reduction in fractional anisotropy, this time in the fronto-limbic circuitry involving the left inferior longitudinal fasciculus, left inferior fronto-occipital fasciculus, and inter-hemispheric fibers of the corpus callosum.^15^ Such effects have been associated with deficits in white matter integrity and one study showed that the myelin-associated water fraction was decreased in the genu of the corpus callosum of first episode patients, whereas chronic patients showed reductions in the same region along with additional changes in the frontal cortex.^16^ Thus, the chronic form of the disease may show changes, which affect a greater number of brain regions.

These studies have led to the hypothesis that brain disconnectivity and the consequential effects on cognitive function are likely to be owing to disruption of axon mylelination by oligodendrocytes. This is likely to be reflected by alterations in the patterns of oligodendrocyte messenger RNA (mRNA) transcripts and proteins. Myelination of axon fibers by oligodendrocytes is essential for rapid conduction of action potentials. This process continues through development into young adulthood and this could be important as this timing coincides with the average age of onset of SCZ.^17^ It is well known that oligodendrocyte dysfunction can lead to disturbances in myelination and consequently to deficient propagation of nerve impulses, compromising cognitive, neural, and glial functions as observed in SCZ.^18^ This is supported by the findings of microscopic stereology and immunohistochemical studies that showed reduced oligodendrocyte density in gray matter of the prefrontal cortex, anterior thalamic nucleus, and the cornuammonis four region of the hippocampus in SCZ patients.^19–23^ Furthermore, electron microscopy studies have revealed dystrophic and degenerative changes of pericapillary oligodendrocytes in the SCZ prefrontal cortex.^24^ However, other studies found no reductions of oligodendrocyte density in white matter,^25,26^ although the two-dimensional assessment of cell density used in some of these cases may have been confounded by volume differences owing to tissue shrinkage that is sometimes associated with formalin fixation.^27^ Although the primary reason for myelin and oligodendrocyte abnormalities in SCZ is not known, similar effects can be found in found in other neurological diseases with signs of inflammatory infiltration and microglial activation.

## The impact of environmental factors leading to oligodendrocyte damage

Along with genetic factors, an array of environmental disturbances can contribute to the development of SCZ.^28^ Epidemiological studies have shown that obstetric complications such as bleeding during pregnancy, abnormal fetal growth, premature labor, or delivery problems are associated with an increased risk of SCZ later in life.^29,30^ All of these scenarios can lead to the development of fetal hypoxia and inflammation,^31,32^ which can become pathological and have a harmful impact on tissue growth. The influence of fetal or perinatal hypoxia on SCZ-like symptoms has been shown in several reports on animal models.^33,34^ Likewise, the potential involvement of inflammation has been supported by a meta-analysis, which found that more than half of the SCZ candidate genes are associated with hypoxia regulation or vascular function.^35^ On the other hand, there are reports showing that hypoxia at low levels is needed for blood vessel formation during embryogenesis^36,37^ and inflammation can protect the immature brain against viral or bacterial infection during pregnancy and may resolve itself without noxious effects.^31^

Hypoxia and inflammation induce changes in gene expression and signaling pathways associated with both physiological and pathological responses throughout the brain, although this occurs predominantly in the microglia. Hypoxia has been shown to activate microglia in the corpus callosum, thereby leading to deficits in myelination and consequently impaired axon functions.^38^ Microglia react rapidly to pathological changes in the brain by producing and releasing various pro-inflammatory cytokines and by generation of free radicals. Other studies have shown that an appropriate interaction between microglia and neurons is required to balance the processes of synaptogenesis and neuronal death during neurodevelopment and brain injury response.^39^

Microglia are mainly activated through inflammation by damage-associated molecular pattern molecules, including ATP high-mobility group box 1 and S100 molecules, as well as pathogen-associated molecular pattern molecules, such as lipopolysaccharide.^40^ Once activated, the microglia themselves can produce both cytokines and growth factors, as well as carry out antigen presentation and phagocytosis.^41^ Thus, microglia can affect other cell types in the area such as the myelinating oligodendrocytes through regulation of inflammation and growth pathways. This is important as some studies have reported increased density of microglia in post-mortem samples from patients with SCZ^42–46^ and patients who had committed suicide during acute psychosis had elevated microglial cell numbers in the anterior cingulate cortex, mediodorsal thalamus, dorsolateral prefrontal cortex, and hippocampus.^44,45^ Also, *in vivo* positron emission tomography imaging analyses demonstrated microglial activation in the hippocampus of patients with SCZ using the benzodiazepine-like ligand [11C] (R)-PK11195,^47^ although treatment of patients with antipsychotics in this study may have been a confounding factor as these drugs are known to decrease cytokine levels.^48^

Taken together, the above findings provide a plausible link between the changes identified by imaging studies regarding white matter density of brains from SCZ patients to alterations in oligodendrocyte functions, such axonal myelination. Studies have shown that cytokines released from activated microglia through hypoxia and inflammation are capable of damaging oligodendrocytes during neurodevelopment stages.^32^ For example, embryonic or neonatal animals treated with specific growth factors or cytokines such as epidermal growth factor, neuregulin 1 (NRG1), interleukin-1, or interleukin-6 exhibit disturbed oligodendrocyte function, abnormal neurotransmission, synaptic loss, and SCZ-like behavioral abnormalities after puberty.^49^ Furthermore, activation of microglia by lipopolysaccharide led to reduced survival of oligodendrocyte precursor cells in oligodendrocyte/microglia co-cultures. Thus, such disturbances could result in impaired connectivity of the developing brain up to the time of adulthood, leading to increased vulnerability of SCZ development. An elegant review has also suggested involvement of microglial or astroglial activation in white matter pathologies in SCZ.^50^ Another study showed that the anti-inflamamtory agent minocycline could be used to inhibit cytokine release and increase survival and proliferation of oligodendrocyte precursor cells in an animal model of hypoxia,^51^ The same study also found that long-term impairment of white matter diffusivity in these animals was attenuated by minocycline, as shown by magnetic resonance imaging/diffusion tensor imaging analysis. Taken together, these findings provide strong evidence that neuroinflammation is associated with oligodendrocyte dysfunction in SCZ, which is likely to lead to alterations in myelination and the white matter tract disturbances associated with disrupted brain connectivity and impaired cognition.

## Differential expression of myelination markers in schizophrenia

In line with the hypothesis of oligodendrocyte dysfunction in SCZ patients and the findings described above, several molecular profiling studies of brain tissues from SCZ patients have now revealed changes in a number of proteins related to myelination. For example, a transcriptomic study carried out in 2001 found altered expression levels of myelination-related mRNAs in post-mortem dorsolateral prefrontal cortex samples, consistent with the oligodendrocyte hypothesis (Table 1).^52^ These findings have been confirmed by other researchers who also investigated myelination-related mRNA levels from post-mortem brains using microarray analyses.^53–55^ In addition to these findings, decreased expression in myelin- and oligodendrocyte-related mRNAs was found in other studies by quantitative PCR and *in situ* hybridization in post-mortem hippocampus and cortical/sub-cortical brain regions from patients with SCZ.^56–59^ More recently, a study employed RNA sequencing in a transcriptomic analysis of post-mortem superior temporal gyrus, which revealed significant alterations of cortical promoter usage and splicing in SCZ patients.^60^ Changes in claudin-11 (CLDN11) mRNA have also been detected in several studies.^55,61–64^ CLDN11 is expressed predominantly by oligodendrocytes and contributes to ~7% of the total myelin mass.^65^ Gow and collaborators reported that claudin-11 is essential for the formation of tight junctions in central nervous system (CNS) myelin through the observation that claudin-11 knockout mice exhibited neurological deficits, such as slowed CNS nerve conduction.^66^

In addition, proteomic studies of post-mortem brain tissues from SCZ patients have been carried out, which identified changes in proteins associated with oligodendrocytes and the process of myelination. In 2004, Prabakaran and collaborators analyzed frontal cortex samples from SCZ patients and controls and found differential expression of transferrin and 2ʹ,3ʹ-cyclic-nucleotide 3ʹ-phosphodiesterase (CNP).^54^ Other studies supported these results through identification of changes in other myelination-related proteins in several different brain regions, such as myelin basic protein (MBP), myelin proteolipid protein (PLP1),^67^ myelin-associated glycoprotein (MAG), and myelin oligodendrocyte glycoprotein (MOG).^16,54,68–75^ The functions of each of these oligodendrocyte proteins are described in more detail in the following sections with a focus on their roles in myelination. It should be noted that there are some discrepancies in the literature regarding the effects on some of these molecules in post-mortem tissues, which could be due to differences in factors such as the methods applied, duration of drug treatment or post-mortem factors. However, another intriguing possibility is that the differences could relate to variations in the brain regions involved and potentially to different studies analyzing different subtypes of the disorder. For example, a recent T1 structural imaging and resting-state functional magnetic resonance imaging scanning study by Chang and co-workers found aberrant bilateral connectivity of default mode network, inferior frontal gyrus, and cerebellum only in patients with auditory verbal hallucinations, whereas disturbances in superior temporal gyrus and precentral gyrus were specific to non-auditory verbal hallucination patients.^51^

### CNP

CNP is an enzyme that catalyses the hydrolysis of a phosphodiester bond in 2ʹ,3ʹ-cyclic phosphate to generate 2ʹ-phosphate.^76,77^ This protein comprises ~4% of the total CNS myelin protein mass and is found in the inner and outer margins of myelin paranodal loops and oligodendrocyte cytoplasm, although it is absent from compact myelin.^78^ CNP undergoes post-translational modification by the addition of isoprenyl and palmitoyllipidic radicals, facilitating its binding to the plasma membranes.^79,80^ In addition to its phosphodiesterase activity, CNP links tubulin to cellular membranes, regulating the microtubule distribution in the cytoplasm. Consequently, it can regulate cellular morphology.^81,82^ Consistent with a potential role in SCZ, two genetic association studies have implicated CNP in SCZ pathogenesis.^83,84^ Likewise, changes in the levels of the CNP protein have been associated with behavioral deficits in mice. Edgar *et al.* ^85^ that CNP knockout mice show decreased emotionality and fear compared with control mice. In addition, we found that it is a potential protein biomarker for SCZ, with altered levels found in some brain regions.^74^

### MBP

MBP has several isoforms, although an 18.5 kDa polypeptide is the major form in adult humans and this is highly conserved in mammals. MBP isolated from brain tissues contains numerous post-translational modifications including deimination, phosphorylation, deamidation, methylation, and N-terminal acylation.^86^ This protein is also attached to the plasma membrane and is responsible for maintaining adhesion of the cytoplasmic surfaces of multi-lamellar compact myelin.^87^ In carrying out this role, MBP interacts with a variety of proteins such as calmodulin, actin, tubulin, and SH3 domain-containing proteins. Thus, it may be involved as a signaling hub in the processes of myelin development and remodeling.^86^ Besides the detection of altered MBP expression in SCZ using transcriptomic^88^ and proteomic analyses, imaging approaches have also found abnormal MBP staining in samples of SCZ brain tissues.^89^ However, another study, which used western blot analysis, found no changes in MBP protein levels in post-mortem brain tissues from patients with SCZ and other psychiatric disorders.^90^ Also, transcriptomic studies found no changes in MBP in post-mortem hippocampus, anterior cingulate cortex, and putamen of patients with SCZ.^62^ In the case of the latter, the discrepancy could be owing to differences in drug treatments and/or potential confounding factors associated with post-mortem tissues such as differences in post-mortem intervals or agonal periods. In addition, differences across studies could be due to the established fact that mRNA and protein levels are not necessarily correlated and deduction from mRNA levels alone is insufficient.^91^ This is why many researchers are turning to protein-based methods as these macromolecules can offer a real-time readout of physiological and biological change. Therefore, further work is essential using proteomic-based methods to establish whether MBP can be a useful biomarker of perturbed myelination in SCZ.

### PLP1

PLP1 is the major protein constituent of compact myelin in the CNS comprising ~45% of the total content.^92^ It has four transmembrane-spanning domains with cytoplasmic N and C termini,^93,94^ and it is highly hydrophobic and contains several covalently bound fatty acid moieties essential for its function. The extracellular loops of PLP1 include four cysteine residues connected through intramolecular disulfide bonds that are crucial for protein folding, dimerization, and cellular trafficking.^95^ One study showed that reduction of PLP1 levels greatly reduced conduction velocity of myelinated axons in mice.^96^ Moreover, these mice displayed anxiety-like behaviors, reduced pre-pulse inhibition, spatial learning deficits, and working memory deficits, as found in SCZ.

### MAG

MAG is a cell adhesion protein found in myelin in both the CNS and peripheral nervous system (PNS) in non-compact regions of the myelin sheath.^97^ This protein is extensively glycosylated on its extracellular loops, which are composed of five tandem immunoglobulin-like domains. The cytoplasmic domains have different intracellular binding sites and properties, suggesting an intracellular signaling function and the capability of interacting with cytoskeletal elements.^98^ Aiming to localize the effects of risk variants in MAG gene on brain morphometry in SCZ patients, Felsky *et al.* ^99^ showed that the temporal and parietal cortices were the areas that were most affected by MAG gene polymorphisms.

### MOG

MOG is also a myelin-associated protein related to the immunoglobulin family and is comprised of 12 isoforms. As the extracellular domain of MOG is capable of undergoing dimerization, Clements *et al.* ^100^ suggested that this assembly could represent a homophilic adhesion complex in myelin sheaths. Other researchers investigated the contribution of genotypic variation of a single-nucleotide polymorphism in *MOG* (rs2857766) to white matter volumes in psychotic disorders.^101^ They found that healthy G-homozygotes of the *MOG* single-nucleotide polymorphism had greater white matter volume in the brainstem and cerebellum compared with those with a psychotic disorder.

### Other proteins

Besides the classical myelin proteins, other proteins related to oligodendocyte function and myelination have been found to be affected in different brain regions from SCZ patients (Table 1). Ermin is an oligodendrocyte-specific protein that appears at a late stage during myelination of mature nerves, and is localized to the outer cytoplasmic lip of the myelin sheath and paranodal loops.^102^ It has been proposed that this protein is involved in regulating cytoskeletal rearrangements by binding F-actin during the late wrapping and/or compaction phases of myelinogenesis.^102^ Gelsolin is a Ca^2+^-dependent actin-binding protein that is found in many types of cells and produced by oligodendrocytes in the CNS. This protein has also been proposed to have role in myelinogenesis it has cleavage, capping, and nucleating activities that might be important in initiating lamellipodia-like protrusive movements of myelin along axonal segments.^103^ Hirakawa *et al.* ^104^ found that the protein hyaluronan and proteoglycan link hyaluprotein 2 (HAPLN2) could have a pivotal role in the formation of the hyaluronan-associated matrix in the CNS, which facilitates neuronal conduction and structural stabilization by mediating binding of versican V2 to hyaluronic acid. Another protein that has been linked to myelination functions is transferrin. This is an iron-binding glycoprotein that controls the levels of free iron in cells, tissues, and body fluids.^105^ In the CNS, transferrin is expressed mainly by oligodendrocytes and found to be differentially expressed in many transcriptomic^60^ and proteomic profiling studies^71,106,107^ of post-mortem brain samples from SCZ patients.

## Myelination pathways mediated by oligodendrocytes

CNS myelination of neuronal axons is mediated by oligodendrocytes in a process involving complex interactions with the extracellular matrix andaxolemma.^108^ The process proceeds in a posterior to anterior gradient across the brain after the neuronal circuitry has been laid down with a peak in early postnatal life and culminating in late-maturing brain structures such as the prefrontal cortex around the time of early adulthood. The key molecules that control myelination are described below.

### NRG1/ErbB

Several isoforms of the NRG1 protein have been shown to be differentially expressed in post-mortem brains of SCZ patients^109^ and mice overexpressing NRG1 types I and III have shown deficits in pre-pulse inhibition, a behavioral test with relevance to symptoms found in SCZ.^110–112^ NRG1 is involved in regulation of glutamatergic receptors, oligodendrocyte proliferation, and myelination,^113^ and can therefore also be linked to brain connectivity. Indeed, myelination is triggered mainly through NRG1/ErbB signaling in oligodendrocytes. The NRG1 family is composed of four proteins belonging to the superfamily of epidermal growth factor-like ligands. Most of these are synthesized as transmembrane precursor polypeptides (pro-NRG1s) with the epidermal growth factor domain located outside the cell. This domain is cleaved by proteases such as tumor necrosis factor-alpha-converting enzyme. This leads to production of mature NRG1s that are soluble, except in the case of NRG1-III. Both the N- and C-terminal regions of NRG1-III are located inside the cell. Thus, NRG1-III may require cell contact to exert its function.^114^

**Figure 1:**
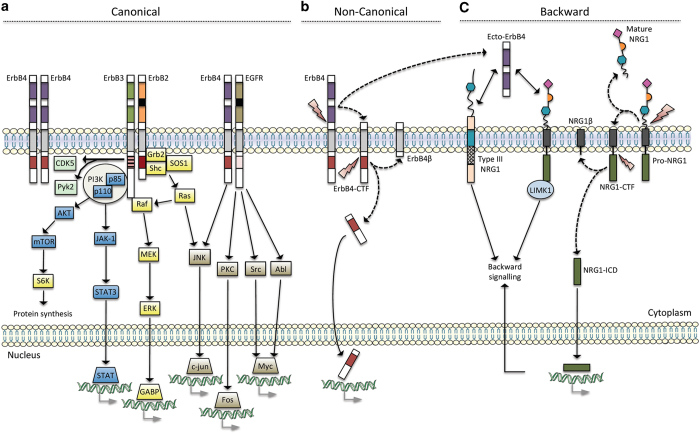
NRG1 and ErbB signaling (adapted from Mei and Xiong^117^). The scheme summarizes the different types of signaling - canonical (a), non-canonical (b) and backward (c) - when NRG1 or ErbB proteins are stimulated. The black boxes indicate that ErbB2 and EGFR do not bind to NRG1. The striped pink box indicates the impaired tyrosine kinase domain of ErbB3 Black arrows indicate downstream signaling pathways when ErbB or NRG1 proteins are activated. Moreover, dashed arrows display the release of protein products from transmembrane proteins that were cleaved by proteases (represented by lightning). Gray arrows indicate gene transcription. NRG1, neuregulin 1.

NRG1 proteins released from neurons bind to ErbB receptors on oligodendrocytes, which initiates several intracellular signaling cascades. The ErbB receptor family is comprised of four structurally related receptor tyrosine kinases (ErbB1–4).^115^ NRG1 binds to ErbB3 and ErbB4 directly. ErbB4 exists as a homodimer^116,117^ and ErbB1 and ErbB2 form heterodimers with ErbB4 and ErbB3, respectively. Dimerization of these proteins triggers NRG1–ErbB signaling in oligodendrocytes as summarized in Figure 1.

Regulation of the NRG1/ErbB pathway in the CNS is achieved mainly through proteolytic cleavage of membrane-bound NRGs by enzymes, such asγ-secretase,^118,119^ β-secretase and a disintegrin, and metalloproteinase domain-containing protein 10 (ADAM10).^120^ Cleavage of NRG1 by ADAM10 and β-secretase releases N-terminal fragments that activate ErbB receptors. However, one study showed that ADAM10 inhibition did not affect normal myelination in a co-culture system, whereas β-secretase inhibition impaired this process.^120^ γ-Secretase can cleave NRG1 and the intracellular domain of the ErbB4 receptor, promoting oligodendrocyte maturation in primary cultures. Moreover, it has been shown that cleavage is likely to block myelination, as inhibitors of this activity accelerate and enhance myelination.^118,121^

### Extracellular matrix components

Several components of the extracellular matrix such as laminin, insulin-like growth factor 1 (IGF-I), and the fibroblast growth factors (FGFs) are essential in the development and function of myelinating cells in the CNS.^122–124^ Laminin receptors such as α6β1-integrin and dystroglycan in oligodendrocyte lineage cells have been shown to mediate oligodendrocyte survival, differentiation, and spatiotemporal targeting in coordination with axonal NRG1 signaling.^125^ Chun *et al.* ^126^ showed that laminin-deficient mice developed demyelinated axons and reduced sheath thickness during the early stages of myelination. In this manner, extracellular laminin could contribute for creating an environment that facilitates myelin production through activation of appropriate signaling pathways.^108^

IGF-I is an anabolic peptide that shares homology with proinsulin.^123^ IGF-I is produced by all major neuronal cell types in the brain and its expression is partly regulated by pituitary growth hormone during development.^127^ IGF-I exerts its action by binding to the IGF-I receptor (IGF-IR), a heterotetrameric glycoprotein composed of two alpha (α) and two beta (β) subunits. The α-subunits constitute the extracellular portion of the receptor with IGF-I-binding sites, whereas the β-subunits span the membrane and initiate intracellular signal transduction via a long intracytoplasmic domain, containing intrinsic tyrosine kinase activity and other critical phosphorylation sites. Studies have shown that the overexpression of IGF-I in the mouse CNS results in increased brain growth, oligodendrocyte number, myelination, and associated myelin protein expression.^128–130^ This is supported by IGF-I knockout mouse studies, which showed reduced numbers of oligodendrocytes and oligodendrocyte precursor cells and decreased myelin-related proteins,^131^ along with decreased neuronal survival.^132^

FGFs are involved in many cell processes and functions, such as proliferation, differentiation, organogenesis, and myelination. FGF-1 and FGF-2 can be produced by neurons and astrocytes,^133,134^ and the levels of both proteins are increased during active myelination.^135^ Consistent with these findings, expression of FGF receptor 1 (FGFR1), FGFR2, and FGFR3 have been detected in oligodendrocytes.^136,137^ These receptors are composed of three immunoglobulin-like domains, a single-transmembrane helical region, and an intracellular domain with tyrosine kinase activity. A recent report showed that oligodendrocytes require FGF receptor signaling to assemble the normal number of myelin membrane wrappings around axons.^124^ However, this is not consistent with the findings of some previous studies, which found that FGF-2 treatment resulted in reduction of myelin formation in rats and cultured neuronal cells.^138,139^

### PI3K/AKT/mTORC signaling

**Figure 2:**
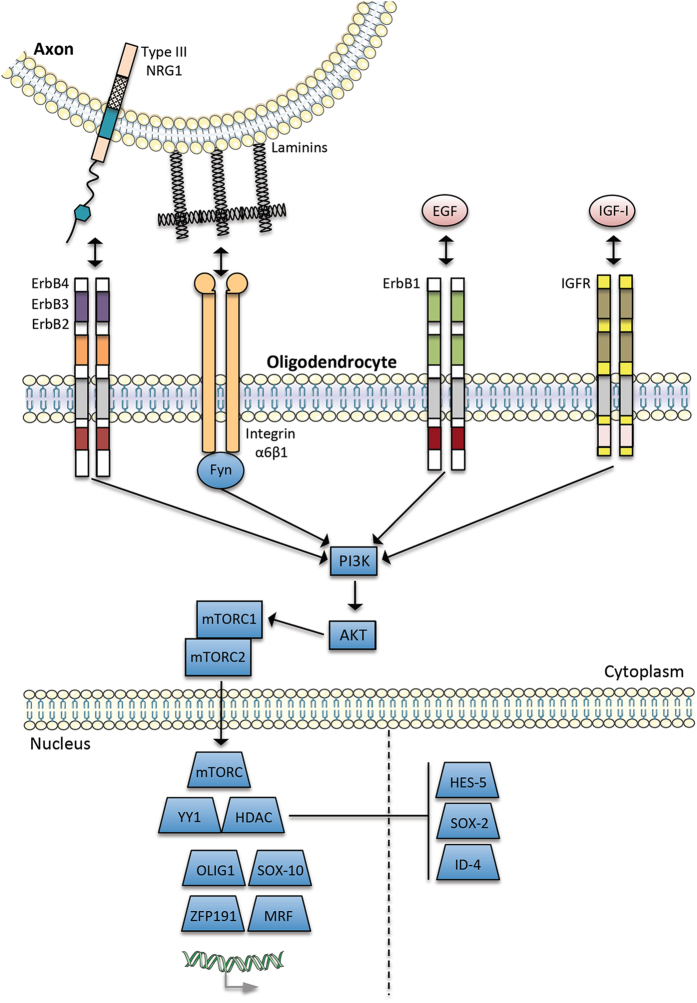
Convergence and integration of external signals in myelination processes by oligodendrocytes (adapted from Taveggia *et al.*^159^). As explained in the text, the PI3K/AKT/mTORC pathway may be the point of convergence and integration of these external signals in myelination process.^159^ The dashed line in the nucleus separates proteins involved in activation and inhibition of myelin gene transcription.

Regarding myelinating factors from the axolemma and extracellular matrix, the phosphatidylinositol 3 kinase/serine–threonine-specific protein kinase/mammalian target of rapamycin complex (PI3K/AKT/mTORc) appears to be a point of convergence in myelination processes (Figure 2). For example, several reports have demonstrated that PI3K/AKT/mTORC signaling is activated by the neuregulins,^140^ integrins,^141^ and IGF-I.^128,142^ Moreover, it has been shown that the expression of constitutively active AKT1 leads to an increase of myelination in mouse oligodendrocytes.^143^ In terminal differentiation of oligodendrocytes, myelin protein and lipid expression can be activated by mTORC1 and mTORC2 complexes induced via PI3K/AKT/mTORC signaling.^144^ A study carried out by Wesseling *et al.* ^145^ identified increased levels of mTOR kinase in the ketamine rat model of SCZ. Apart from effects on myelination, the mTOR kinase is also crucial for regulating expression of proteins involved in synaptic plasticity.^146^ In addition, mTOR signaling has been reported disrupted in SCZ patients^147^ and reduced levels of synaptic proteins normally induced by mTOR signaling have been reported in the prefrontal cortex of depressed patients.^148^ Therefore, these findings support the possibility of targeting components of the mTOR signaling pathway as a potential new approach in the development of antipsychotic drugs. Other nuclear proteins involved in activation and inhibition of myelin gene transcription are also shown in Figure 2.

## Future work: identification of novel targets for schizophrenia associated with oligodendrocyte and myelin function

Considering that proteins do not work alone but rather as multi-protein complexes, pathways, or signaling cascades, further studies should be carried out to map the protein and small molecule networks associated with the oligendrocyte-related proteins discussed in this review. This could be achieved using laboratory techniques such as tandem affinity purification, which employs a tagged protein bait for co-purification of interacting proteins in cells and mass spectrometry for identification.^149^ In addition, *in silico* methods could be applied such as Ingenuity Pathway Analysis^150^ or GeneMANIA,^151^ which can identify potential interaction partners in protein networks by superimposing laboratory data on to pre-existing networks in interaction databases. Subsequently, further mapping studies known as ‘pathway walking’ can be performed as more SCZ-related molecules are identified, thereby extending networks to include key regulatory points, which may be druggable.

As inflammation and immune activation have been implicated in the development and progression of SCZ, it follows that compounds capable of normalizing such imbalances may be helpful in treating the disease. For example, a study using a rat model of impaired myelin production found that oligodendrocytes recovered their function after restoration of immunoglobulin Fc receptor gamma/Fyn signaling.^152^ Another study showed that the antibiotic minocyclin was capable of reducing oligodendrocyte damage caused by gamma-interferon-stimulated microglia in a cell co-culture investigation^153^ and the same compound was used inhibit cytokine release and increase oligodendrocyte precursor cell survival in a hypoxia-based animal model, as described above.^51^ A recent clinical study found that adjunctive minocycline with clozapine treatment helped to relieve impaired working memory, avolition, and depression/anxiety in chronic SCZ patients with persistent symptoms.^154^ On the basis of studies such as these, researchers have suggested that the best approach would be to combine the use of anti-inflammatory substances with standard antipsychotic treatment for improved treatment of those SCZ patients who also display an inflammatory phenotype.^155^ Furthermore, growth factors, such as IGF-I, are also under consideration as new therapeutic candidates, as the IGF pathway is known to be involved in the regulation of oligodendrocyte development and repair.^156^ In line with this, a recent study published in *Nature* showed that IGF-I treatment restored synaptic deficits in neurons from 22q11.2 deletion patients, a syndrome characterized by an increased risk of SCZ and other psychiatric conditions.^156^ According to some studies, the initiation of treatment is likely to be more effective at the onset of symptoms,^157^ suggesting that it may be more appropriate for testing in patients with first episode psychosis and as an adjuvant treatment to antipsychotic agents, as described above for the anti-inflammatory agents.

## Final remarks

The study of SCZ is challenging owing to its complex nature. However, breakthroughs are essential to potential shed light on new possible treatment avenues. Numerous clinical studies using imaging techniques have reported white matter changes in SCZ indicative of perturbations in connectivity within and across different brain regions. Furthermore, recent molecular studies, such as transcriptomic and proteomic profiling analyses of brain tissues and cell culture models have implicated oligodendrocyte and myelination dysfunction as significant features of the disease.^158^ The present review has linked imaging and molecular data on these effects in SCZ, providing an impetus for further studies in this area. It is only through an increased understanding of the disease pathways that much-needed novel drug targets can be identified.

## Figures and Tables

**Table 1:** Classical myelin proteins and most oligendrocyte-related proteins differential expressed in schizophrenia

*Proteins*	*UniProt ID, human*	*Cellular location*	*Gene expression reference*	*Proteome reference*
2,3-Cyclic-nucleotide 3-phosphodiesterase (CNP)	P09543.2	Plasma membrane	52, 56, 60–62, 64	16, 54, 70, 71
Claudin-11 (CLDN11)	O75508.2	Tight junction	55, 61–63	—
Myelin basic protein (MBP)	P02686	Plasma membrane	55, 60, 88	68, 69, 71, 74
Myelin proteolipid protein (PLP1)	P60201.2	Plasma membrane	55, 67	72
Myelin-associated glycoprotein (MAG)	P20916.1	Plasma membrane	52, 53, 55, 56, 60–62, 67	73
Myelin oligodendrocyte glycoprotein (MOG)	Q16653.1	Plasma membrane	55, 56, 60, 61, 75	70, 71, 74
Hyaluronan and proteoglycan link hyaluprotein 2 (HAPLN2)	Q9GZV7	Secreted	—	71
Ermin (ERMN)	Q8TAM6	Cytoskeleton	—	71
Gelsolin (GSN)	P06396	Cytoskeleton	—	54
Transferrin (TRF)	P02787	Cytoplasm	52, 55, 60, 61, 64, 67, 75	54, 69, 106, 107

## Notes

AS was an honorary speaker for TAD Pharma and Roche and has been a member of advisory boards for Roche. PF has been an honorary speaker for AstraZeneca, Bristol Myers Squibb, Eli Lilly, Essex, GE Healthcare, GlaxoSmithKline, Janssen Cilag, Lundbeck, Otsuka, Pfizer, Servier, and Takeda. During the past 5 years, but not presently, PF has been a member of the advisory boards of Janssen Cilag, AstraZeneca, Eli Lilly, and Lundbeck. The remaining authors declare no conflict of interest.
